# Characterization of pig saliva as the major natural habitat of *Streptococcus suis* by analyzing oral, fecal, vaginal, and environmental microbiota

**DOI:** 10.1371/journal.pone.0215983

**Published:** 2019-04-24

**Authors:** Kazunori Murase, Takayasu Watanabe, Sakura Arai, Hyunjung Kim, Mari Tohya, Kasumi Ishida-Kuroki, Tấn Hùng Võ, Thị Phương Bình Nguyễn, Ichiro Nakagawa, Ro Osawa, Ngọc Hải Nguyễn, Tsutomu Sekizaki

**Affiliations:** 1 Department of Microbiology, Graduate School of Medicine, Kyoto University, Kyoto, Japan; 2 Research Center for Food Safety, Graduate School of Agricultural and Life Sciences, the University of Tokyo, Tokyo, Japan; 3 Veterinary Diagnosis Laboratory, Faculty of Animal Science and Veterinary Medicine, Nong Lam University, Ho Chi Minh, Vietnam; 4 Department of Bioresource Sciences, Graduate School of Agricultural Sciences, Kobe University, Hyogo, Japan; 5 Faculty of Animal Science and Veterinary Medicine, Nong Lam University, Ho Chi Minh, Vietnam; Universite Paris-Sud, FRANCE

## Abstract

It is generally difficult to specify the sources of infection by which domestic animals may acquire pathogens. Through 16S rRNA gene amplicon sequencing, we compared the composition of microbiota in the saliva, vaginal mucus, and feces of pigs, and in swabs of feeder troughs and water dispensers collected from pig farms in Vietnam. The composition of the microbiota differed between samples in each sample group. *Streptococcus*, *Actinobacillus*, *Moraxella*, and *Rothia* were the most abundant genera and significantly discriminative in saliva samples, regardless of the plasticity and changeability of the composition of microbiota in saliva. Moreover, species assignment of the genus *Streptococcus* revealed that *Streptococcus suis* was exceptional in the salivary microbiota, due to being most abundant among the streptococcal species and sharing estimated proportions of 5.7%–9.4% of the total bacteria in saliva. Thus, pig oral microbiota showed unique characteristics in which the major species was the pig pathogen. On the other hand, β-diversity analysis showed that the microbiota in saliva was distinct from those in the others. From the above results, pig saliva was shown to be the major natural habitat of *S*. *suis*, and is suggested to be the most probable source of *S*. *suis* infection.

## Introduction

*Streptococcus suis* (*S*. *suis*) is a major swine pathogen that causes various diseases such as meningitis, septicemia, and endocarditis, which result in large economic losses in the pig industry [1, 2]. This bacterium is also an emerging zoonotic pathogen because life-threatening and fatal *S*. *suis* infections have been reported in people engaged in slaughtering pigs and processing raw pork [3, 4]. In East and Southeast Asia, *S*. *suis* is a major public health concern. In China, large outbreaks of human infection occurred in 1998 and 2005, and caused severe diseases such as meningitis and septicemia or toxic shock-like syndrome [5]. In the ranking of pathogens causing adult infectious meningitis, *S*. *suis* ranks first in Vietnam and ranks second in Thailand [6, 7]. Therefore, it is important to investigate the prevalence of *S*. *suis* among pigs in these areas.

Despite many efforts to isolate *S*. *suis* from the organs of healthy and diseased pigs, the main source of infection has not been fully elucidated. Previous studies showed that 40%–80% of healthy pigs carry *S*. *suis* in their nasal cavities, tonsils, vagina, and digestive tract. *S*. *suis* may colonize pigs through vertical transmission during parturition or through horizontal transmission by aerosols [1, 8–10]. Experimental exposure to airborne *S*. *suis* is a proven cause of infection [11]. It is also possible that *S*. *suis* can infect through the fecal-oral route. Such pigs occasionally show symptoms of *S*. *suis* infection; pigs at the post-weaning stage, in particular, are often slaughtered by the disease.

The sources of transmission in *S*. *suis* infection have been investigated by bacterial culture used in combination with serotyping, and restriction fragment length polymorphism, or PCR detection of *S*. *suis* [12–15]. By means of quantitative PCR, we recently showed that 100% of saliva samples from healthy pigs in Japan contained *S*. *suis*, whereas not all samples of feces, vaginal mucus, and swabs of feed troughs and water dispensers contained *S*. *suis* [16]. Comparison of the amount of *S*. *suis* in saliva samples at various growth stages suggested that the saliva of sows was the most probable reservoir and the source of infection for pigs. However, these techniques do not allow the ability to analyze whole microbial populations and can only provide information that is limited to a specific marker organism.

With the recent advances in high-throughput sequencing techniques, 16S rRNA gene amplicon sequencing is used to display the microbiota in biotic and abiotic samples. The 16S rRNA gene amplicon sequencing provides higher resolution of the taxonomical data to the genera level. However, classifying the microbial community based on a single gene or on part of the 16S rRNA gene could limit the results to a certain confidence level, especially when one genus involves many species that are closely related to each other. The genus *Streptococcus* is an example. For the discrimination of operational taxonomic units (OTUs) in the genus *Streptococcus* at the species level, a customized database depositing the sequence data of the respective genus is needed. Alternative proficiency of 16S rRNA gene amplicon sequencing is the availability of various bioinformatic methods for comparative analyses such as α diversity for comparing the diversity and richness involved in the microbiota.

In this study, 16S rRNA gene amplicon sequencing was performed for samples of pig saliva, feces, and vaginal mucus, and from swabs of feeder troughs and water dispensers used in pig farms in Vietnam. The sequencing allowed us to compare the composition of microbiota in these samples. Construction of an original database comprising all available sequences in the genus *Streptococcus* made it possible to estimate the relative amounts of *S*. *suis* in the microbiota from different sources. Furthermore, several bioinformatic analyses provided interesting insights into the ecological nature of *S*. *suis* in pig bodies and in their living environments.

## Materials and methods

### Ethics statement

All animal experiments described in this study were conformed to the Guidelines for Animal Experiments of the University of Tokyo (Tokyo, Japan) and were approved by the Animal Research Committee of the University of Tokyo (approval no. P16-289).

### Animals and sample collection

From October 2015 to November 2015, samples of saliva, feces, and vaginal mucus (i.e., biological samples) were collected from sows and piglets on two pig farms in Vietnam. Swab specimens of feeder troughs and water dispensers (i.e., environmental samples) on these two farms were also collected (S1 Table). Owner consent was provided for the use of the animals and collection of samples. Piglets were categorized into three growth stages, based on age: suckling piglets, which were within 20 days old; post-weaning piglets, which were from weaning to 30 days old; and growing piglets, which were 60–90 days old. Each biological sample was collected from the individual pig without known parental or blood relationship to each other except for the following cases; several pairs of salivary, fecal, and vaginal mucus samples were collected from the same pigs, and other several samples were collected from sows and their piglets or from piglets which were born from the same sow (S1 Table). Antimicrobials were not used for treating diseases, but were added in the feed at all farms enrolled in the study. All pigs appeared to be healthy; no animal had signs of disease. Saliva samples were collected as described previously [16]. Briefly, the saliva samples were collected by wiping the surface of porcine oral cavity with cotton swabs. The feces samples were collected with Fisherbrand disposable sterile spoons (Thermo Fisher Scientific). The samples from vagina, feeder trough, and water dispenser were collected with BD BBV CultureSwab EZ Ⅱ (Becton, Dickinson and Company, Milano, Italy). All samples were immersed in a RNAlater Stabilizing Solution (Thermo Fisher Scientific, Waltham, MA, USA) in conical tubes to prevent bacterial growth and degradation of the DNA. The tubes were stored at –20°C until use.

### Extraction of DNA

Frozen samples were thawed and centrifuged at 5,500 rpm (6,230 × *g*) for 10 minutes at 4°C. The pellet in each tube was washed twice with sterile 0.85% saline solution and then resuspended in 1 mL of the saline solution. An aliquot (800 μL) was centrifuged again; the pellet was resuspended in 350 μL of solution 1 from the PowerBiofilm DNA Isolation Kit (Qiagen, Hilden, Germany) and used for the extraction of total DNA in accordance with the manufacturer’s instructions, but with a few modifications. Instead of using beads provided in the kit, 400 μL of 0.5-mm-diameter zirconia beads and two 5-mm-diameter zirconia beads were used to crush bacterial cells. Bacteria were crushed by using Beads Crusher μT-01 (Taitec Corp., Tokyo, Japan) at 4,600 rpm for seven repeats of 60-second run with 60-second intervals. The total DNA was eluted in 100 μL of the elution buffer provided in the kit and stored at –20°C until use. The concentration of DNA was measured with a Quantus Fluorometer (Promega, Madison, WI, USA) with the QuantiFluor dsDNA System (Promega) according to the manufacturer’s instructions. The DNA quality was verified by measuring the ratio of the absorbance at 260 nm and 280 nm (ratio, 1.8–2.0) with a NanoDrop 1000 Spectrophotometer (Thermo Fisher Scientific).

### qPCR

The qPCRs assay and the calculation of cell numbers have been described previously [16]. Briefly, each qPCR assay for total bacteria (qPCR_TB_) or *S*. *suis* (qPCR_SS_) was performed in a total volume of 20 μl, which contained 2 μl of DNA template, 0.4 μM each primer (0.2 μM each primer for qPCR_TB_ assay), 0.2 μM probe, 1× ROX reference dye, and 1× THUNDERBIRD Probe qPCR Mix (Toyobo, Osaka, Japan). The qPCR assay conditions were 1 minute at 95°C, 40 cycles of 15 seconds at 95°C and 1 minute at 60°C. Distilled water was used as the template for negative control, and all reactions were performed in triplicate. For the standard curve, data obtained for the qPCR assay with the serial dilution templates were plotted against cell numbers, which were calculated from the DNA concentrations and the genome size. The cell numbers of *S*. *suis* were estimated from the standard curve. The cell numbers of total bacteria in the sample were estimated by use of *Escherichia coli*; therefore, we converted the number of *E*. *coli* into the number of total bacteria as follows: number of total bacteria = number of *E*. *coli* x 7/4.1858. The values 7 and 4.1858 represent the mean copy numbers of the 16S rRNA gene of *E*. *coli* and total bacteria, respectively. The mean copy number for total bacteria was estimated from 1,690 publicly available complete bacterial genomes [17].

### PCR amplification and 16S rRNA gene amplicon sequencing

The V3-V4 hypervariable regions of 16S rRNA genes were amplified by PCR from the DNA extracted from the samples. Before PCR amplification, the amount of the total bacterial genome DNA contained in the extracted DNA was estimated by the qPCR_TB_. The bacterial genomic DNA of 12.5 ng equivalent to 3.2 × 10^6^ copies of the bacterial genome was amplified in a 25-μL reaction buffer containing 12.5 μL of 2× KAPA HiFi HotStart Ready Mix (Kapa Biosystems, Woburn, MA, USA), 2.5 μL of template DNA, and 5 μL each (i.e., 1 μM of each) of the forward primers and reverse primers. The primers with overhang adapter sequences used to amplify the V3-V4 region of the 16S rRNA genes were S-D-Bact-0341-b-S-17 (5´-TCGTCGGCAGCGTCAGATGTGTATAAGAGACAGCCTACGGGNGGCWGCAG-3´) and S-D-Bact-0785-a-A-21 (5´-GTCTCGTGGGCTCGGAGATGTGTATAAGAGACAGGACTACHVGGGTATCTAATCC-3´). The PCR program was as follows: 95°C for 3 minutes; 25 cycles of 95°C for 30 seconds, 55°C for 30 seconds, and 72°C for 30 seconds; and a final extension at 72°C for 5 minutes.

The quality of the PCR products was ensured using the Agilent 2100 Bioanalyzer (Agilent Technologies Japan, Tokyo, Japan) in accordance with the manufacturer’s instructions. The products were then cleaned up by using Agencourt AMPure XP beads (Beckman Coulter Inc., Brea, CA, USA). The dual indices and sequencing adapters were attached by the subsequent PCR using the Nextera XT Index Kit (Illumina, San Diego, CA, USA). The indexed products were further purified by AMPure XP beads and ensured by the Bioanalyzer. The libraries prepared were quantified by qPCR with the Library Quantification Kit for Illumina (Kapa Biosciences). Libraries of equal molars were pooled and diluted in a hybridization buffer. The pooled libraries were heat-denatured and mixed with 50% (v/v) of the Illumina PhiX control DNA before loading the sequencer. The amplicons were sequenced using a 2 × 301 paired-end method with MiSeq Reagent Kit v3 on the Illumina MiSeq platform, based on the manufacturer’s instructions.

### Bioinformatics processing of 16S rRNA gene amplicon sequence data

Demultiplexed forward (R1) and reverse (R2) reads were processed using Illinois-Mayo Taxon Organization from RNA Dataset Operations (IM-TORNADO) pipeline [18]. The software Trimmomatic was used in the pipeline to trim the forward and the reverse primers from the 5´ end of R1 and R2, respectively, and the adaptor sequences from the 3´ ends of the reads [19]. The resultant reads were merged and then processed to trim low-quality bases, to remove with ambiguous bases, and to dereplicate building clusters of reads with 100% similarity and annotated with cluster size. For ensuring the use of high quality reads when assigning OTU representation, singletons and reads shorter than the cutoff length were discarded. Reads were sorted by cluster size and processed in USEARCH using the UPARSE algorithm to detect the OTU representatives using *de novo* OTU picking strategy. During this step, we also removed chimeric reads, which resulted in a set of OTU representatives with very high sequence quality. The OTUs were picked and identified at 97% similarity using the RDP (version 11; Ribosomal Database Project, Michigan State University, East Lansing, MI, USA) [20] as the reference database. The IM-TORNADO pipeline also was used to assign the taxonomy of the OTUs by mothur. The number of sequences was normalized to 43,000 for each sample by random subsampling and used for the following analyses. The individual sample richness and diversity of biological and environmental samples were measured by bootstrapping random sequences from the sequences in each sample for 50 times.

### Species assignment of OTUs involved in the genus *Streptococcus*

Customized database for species assignment in *Streptococcus* was constructed by retrieving the 16S rRNA gene sequences from the following sequence data sources: (i) complete genome sequences of 123 strains of 29 *Streptococcus* species in the GenBank [21]; (ii) draft genome sequences of 8 *S*. *parasuis* strains [22]; (iii) 16S rRNA sequences of 29 *S*. *suis*, 13 *S*. *parasuis*, 2 *S*. *orisratti*, and 1 *S*. *ruminantium* strains [23]; and (iv) 16S rRNA gene sequences of 4,596 streptococcal strains in the RDP (last accessed in January 2016). These sequences were dereplicated using Cluster Database at High Identity with Tolerance (CD-HIT) program [24] with the sequence identity threshold of 1.0, and were finally clustered into 909 groups. These groups were taxonomically assigned using the following criteria with consideration of phylogenetic relationship: (i) if all sequences in a group were derived from a single species, the group was assigned as that species; (ii) if the sequences in a group were derived from more than one species, and the sequences of the most predominant species accounted for more than 80% of the total sequences in the group, the group was assigned as the most predominant species; and (iii) if a group did not correspond to the previous two criteria, the group was assigned as an unknown species (S2 Table). The maximum-likelihood phylogenetic tree of 909 representative sequences was reconstructed using FastTree [25] with default parameter (S1 Fig). The preprocessed reads from saliva samples were aligned with the representative sequences of all groups in our customized database by using the VSEARCH program [26] under the threshold of 98.5% similarity. Bacterial composition at the species level in *Streptococcus* was presented in visual form by a pie chart.

### Computational analyses of relative gene abundance

The Chao1, Equitability, Shannon, and Simpson indices and Good’s coverage (i.e., α-diversity indices), and common OTUs were calculated by Quantitative Insights Into Microbial Ecology (QIIME; v1.9.1) [27]. A Venn diagram was generated to compare OTUs between groups using R [28]. Genera distributions in the microbiota in each sample group were calculated using QIIME and are presented as the relative abundance (%) in a stacked bar chart. The top three genera in each sample group are presented, based on the data calculated by QIIME. Bacterial taxa that were significantly enriched in a certain sample group were extracted by Linear discriminant analysis (LDA) effect size (LEfSe) [29]. In the LEfSe analysis, the Kruskal-Wallis test was used to obtain an LDA score under the threshold of >4.0 (*p*<0.01) with all-against-all multiclass analysis in the online interface Galaxy (http://huttenhower.sph.harvard.edu/lefse/). Alpha diversity was estimated using the following indices: the number of observed OTUs, Good’s coverage, Chao1 richness [30], Simpson diversity index [31], and Shannon diversity index [32]. A rarefaction curve was constructed from the number of OTUs in each sample. Beta diversity was estimated using Principal coordinate analysis (PCoA), based on the unweighted UniFrac distance matrix [33]. Hierarchical cluster analysis was conducted using the unweighted pair-group method with arithmetic mean on the QIIME platform. Spearman’s rank correlation coefficient was calculated for all bacterial genera against the *S*. *suis* using MicrobiomeAnalyst [34], under the thresholds of 𝜌≥0.4, *p*<0.01 or *p*<0.05, and a false discovery rate<0.1. Statistical significance of the correlation coefficient was evaluated by the *t* test. The bacterial genera extracted were further searched against the database to assign bacterial species by using the BLASTN program (National Center for Biotechnology Information, Bethesda, MD, USA).

## Results

### DNA sequence data and the abundance of OTUs inferred from the 16S rRNA gene

A total of 3,385,317 sequence reads of the V3-V4 region of 16S rRNA gene were generated from 81 samples (45 saliva samples, 9 feces samples, 6 vaginal mucus samples, and swabs of 9 feeder troughs and 12 water dispensers) with an average of 74,534–89,481 sequence reads for each sample. Good’s coverage was more than 99.5%, which suggested that the reads obtained for the samples represented a sufficient number of sequences for the analysis of the composition of microbiota. Based on 97% sequence similarity, an average of 558, 885, 665, 698, and 446 OTUs were obtained from samples of saliva, feces, vaginal mucus, swabs of feeder troughs and water dispensers, respectively. The lowest level of taxon abundance for each OTU was determined by combining homologous sequence alignment and clustering based on information extracted from the RDP classifier. As a result, 22.7% of sequences could not be assigned at the genus level. The Shannon diversity curves for all samples plateaued, although the individual rarefaction curves did not reach the saturation phase (S2 Fig). This finding suggested that increasing the sequencing depth would possibly identify more new phylogenetic types; however, most of the microbial diversity was considered to be captured by the current analysis.

**Fig 1:**
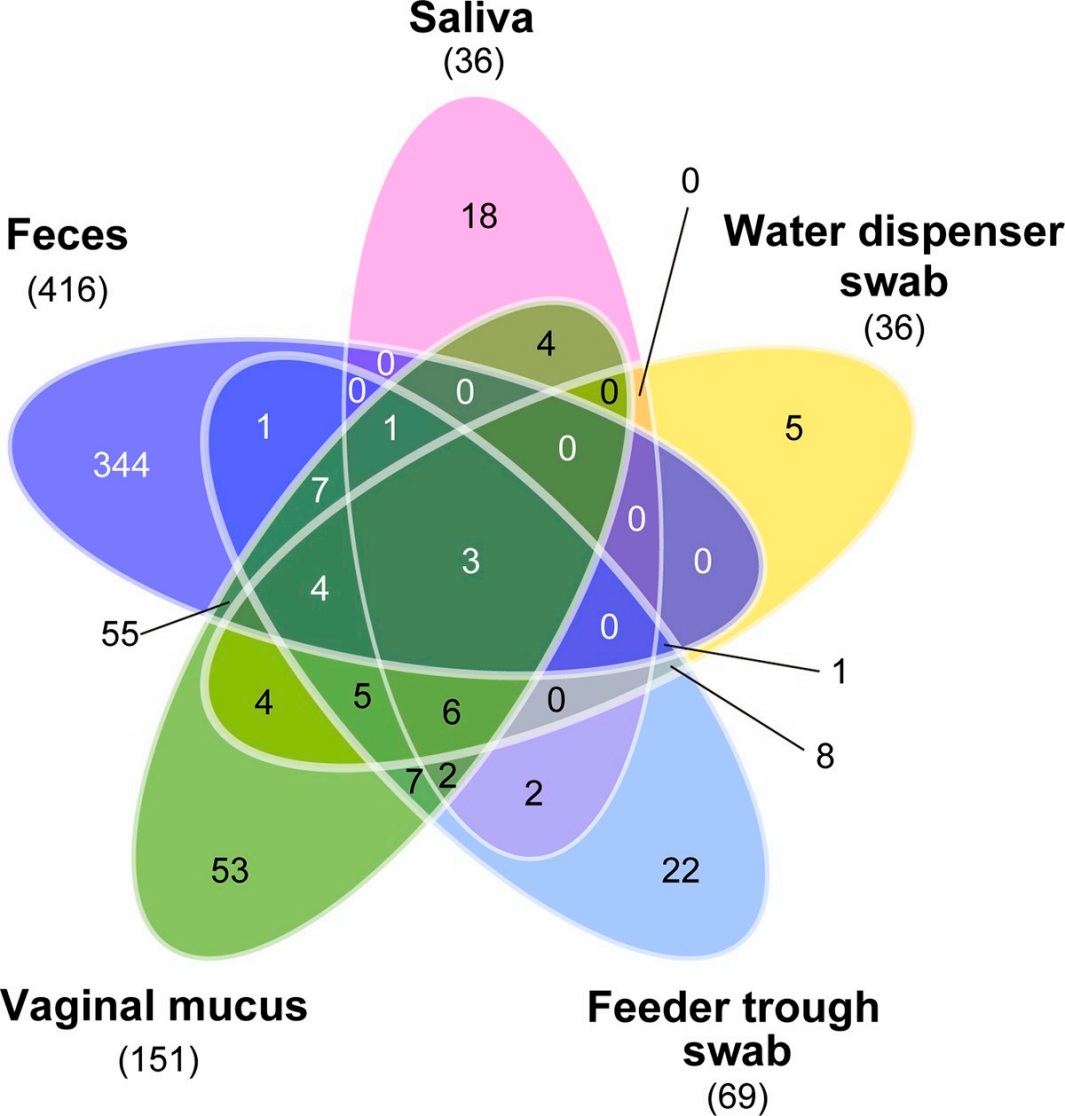
Comparison of OTUs between the five sample groups. The Venn diagram presents the common and unique OTUs among the five sample groups. The number of common OTUs is indicated in parenthesis under each sample name.

The term “richness” refers to the number of species in a microbiota, whereas “diversity” refers to the number of species and evenness of distribution of the bacterial species. Microbial diversity within a local community (i.e., α diversity) is measured by either the richness (i.e., Chao1) or by diversity indices such as the Shannon index or Simpson index. The α diversity and richness of biological and environmental samples are presented in Table 1. A large number of OTUs and higher values for the Shannon and Simpson indices in feces samples indicated a more diversified microbial community. Feces samples similarly exhibited higher species richness, as measured by the Chao 1 index, than did the other samples. The number of OTUs that appeared in all samples in each group—hereafter referred to as “common OTUs”—were 36, 416, 151, 69, and 36 in saliva, feces, vaginal mucus, and in swabs of feeder troughs and water dispensers, respectively (Table 1). This finding also indicated that the microbiota of feces was the most diversified. By contrast, the number of common OTUs was lowest in saliva and in swabs of water dispensers. Only three OTUs were commonly present in all sample groups, whereas 18, 344, 53, 22, and 5 OTUs were uniquely identified in saliva, feces, vaginal mucus, and in swabs of feeder troughs and water dispensers, respectively (Fig 1).

**Table 1:** The α diversity and richness of the 16S rRNA gene.

Sample type	Chao1	Equitability	Shannon	Simpson	Common OTU
Biological					
- Saliva	736.1 ± 238.7	0.61 ± 0.07	5.5 ± 0.85	0.93 ± 0.04	36
- Feces	1,033.3 ± 82.8	0.66 ± 0.03	6.5 ± 0.38	0.96 ± 0.01	416
- Vaginal mucus	843.1 ± 303.1	0.59 ± 0.06	5.5 ± 0.86	0.94 ± 0.03	151
Environment					
- Feeder trough swab	925.6 ± 366.3	0.60 ± 0.11	5.6 ± 1.35	0.92 ± 0.09	69
- Water dispenser swab	615.1 ± 356.3	0.62 ± 0.07	5.3 ± 0.97	0.93 ± 0.05	36

The data are presented as the mean ± the standard deviation. Alpha-diversity indices were measured by the subsampled 43,000 reads of each sample.

### Characterization of the microbiota

**Fig 2:**
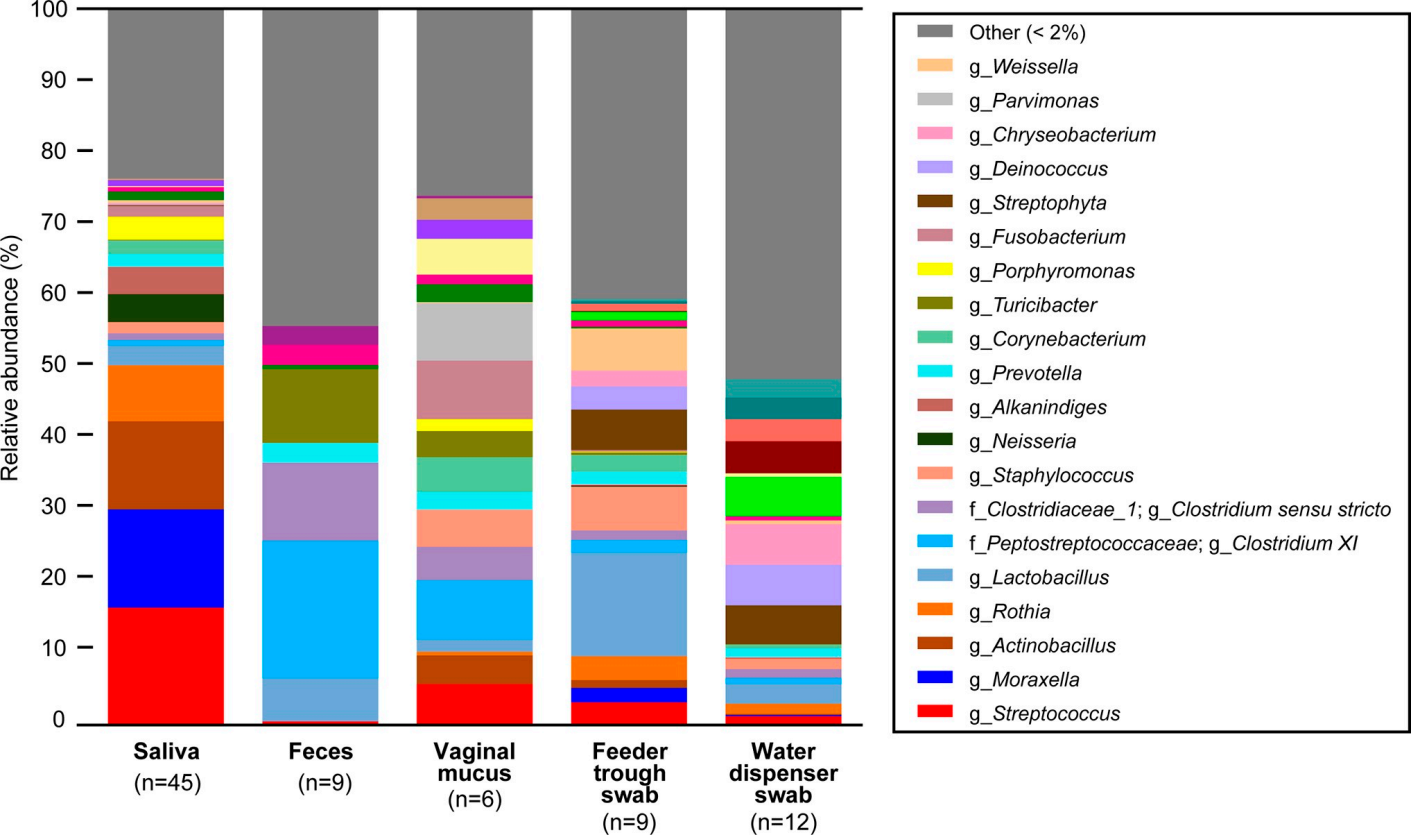
Genera distribution of the microbiota in the biological and environmental samples from pig farms. Only the bacterial genera share more than 2% relative abundance, which is indicated by different colors. The genera that share less than 2% are collected and then indicated by gray bars. Prefixes before the bacterial names indicate the following ranks: “f” for family, and “g” for genus.

**Fig 3:**
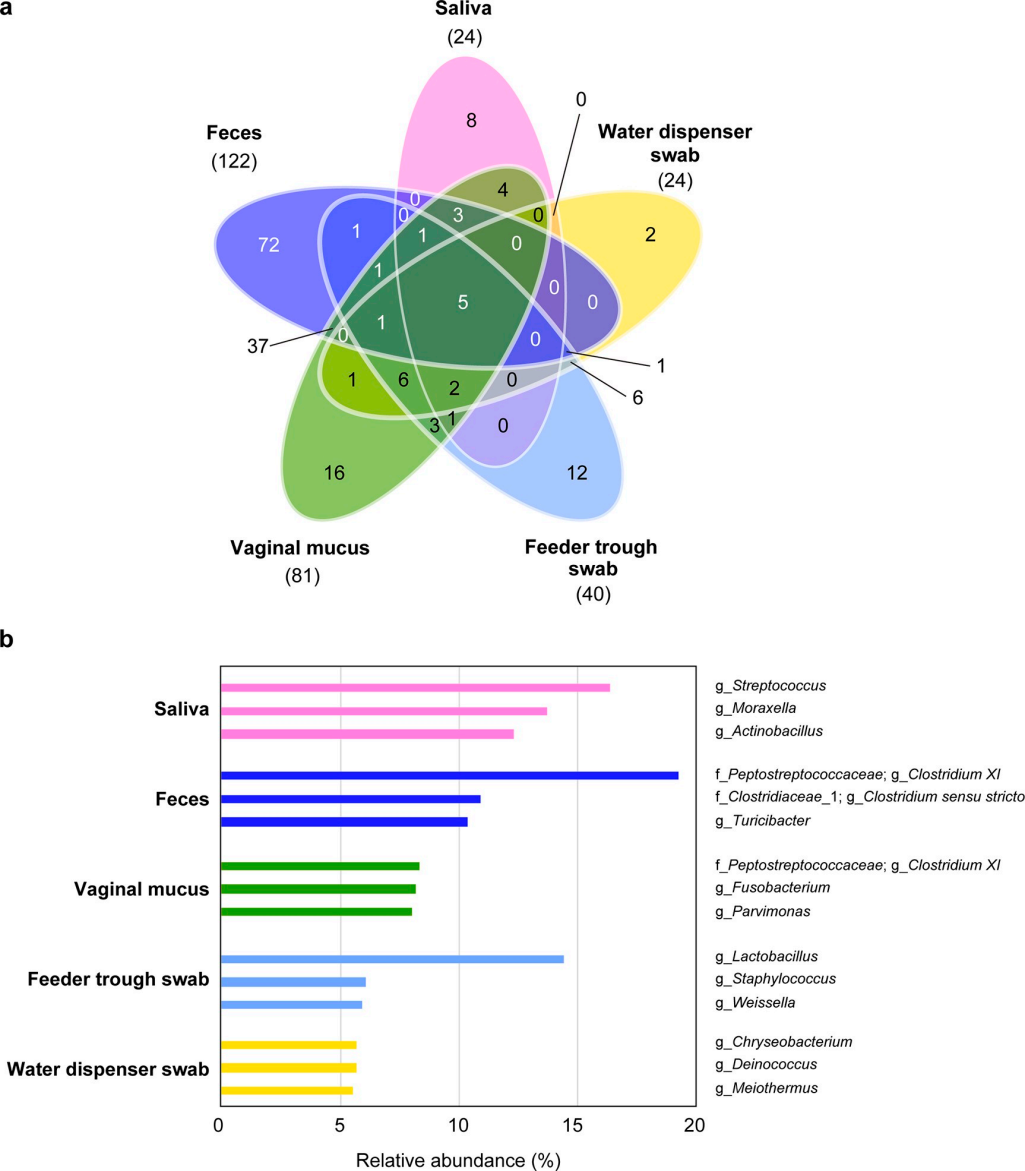
Common, unique and top three genera. (a) The Venn diagram presents the common and unique genera among the five sample groups. The number of common genera that were present in all samples in each group is indicated in parenthesis under each sample’s name. (b) The top three genera identified in the five sample groups.

The composition of the microbiota, the major genera, and their proportions in saliva, feces, vaginal mucus, swabs of feeder troughs and water dispensers seemed to differ from each other. In particular, the microbiota characteristics of saliva were uniquely different from those of the other samples (Fig 2). The four genera *Streptococcus*, *Moraxella*, *Actinobacillus*, and *Rothia* shared nearly 50% of the microbiota in saliva. Only five genera were commonly present in all five sample groups, whereas 8, 72, 16, 12, and 2 genera were uniquely identified in saliva, feces, and vaginal mucus, and in swabs of feeder troughs and water dispensers, respectively (Fig 3A).

The top three genera in the saliva samples were *Streptococcus*, *Moraxella*, and *Actinobacillus* (shared 12.3%–16.3%). In the other sample groups, however, these three genera did not appear in the top three genera. Their abundances in the other sample groups were very low (Fig 3B) except for vaginal mucus and swabs of feeder troughs in which these three genera shared 1.1%–5.7% (Fig 2).

**Fig 4:**
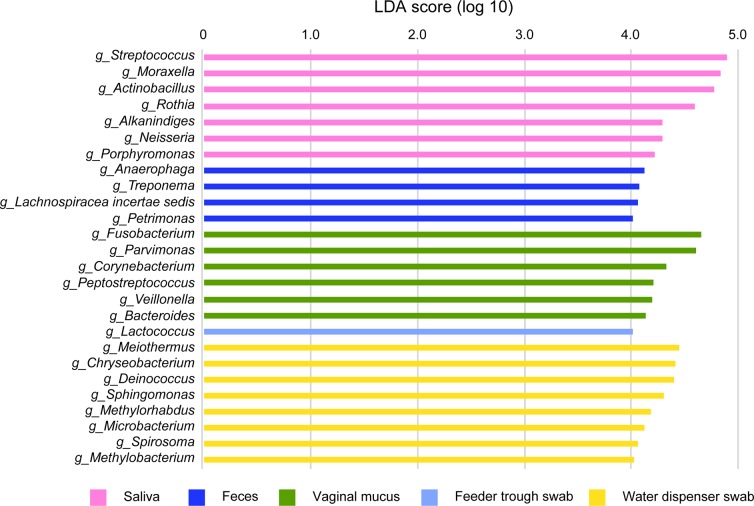
Results of the LEfSe analysis show significantly discriminative genera among the five sample groups. Genera with an LDA score >4.0 (*p*<0.01) are depicted, based on their scores. Prefixes of the bacterial names indicate the following ranks: “o” for order, “f” for family, and “g” for genus.

LEfSe analysis can highlight the major bacterial taxa in a sample [29]. The LEfSe analysis revealed seven significantly discriminative genera in the saliva samples (Fig 4). Among these seven genera, *Streptococcus*, *Moraxella*, *Actinobacillus*, and *Rothia* recorded high LDA score in the saliva samples, and three of these four genera appeared in the top three genera in the saliva samples. On the other hand, four, six, one, and eight distinct genera were significantly more abundant in feces and vaginal mucus, and in swabs of feeder troughs and water dispensers, respectively (Fig 4). Among these genera, *Fusobacterium* and *Parvimonas* in vaginal mucus and *Meiothermus*, *Chryseobvacterium*, and *Deinococcus* in water dispenser swabs were in the top three genera in each sample group.

**Fig 5:**
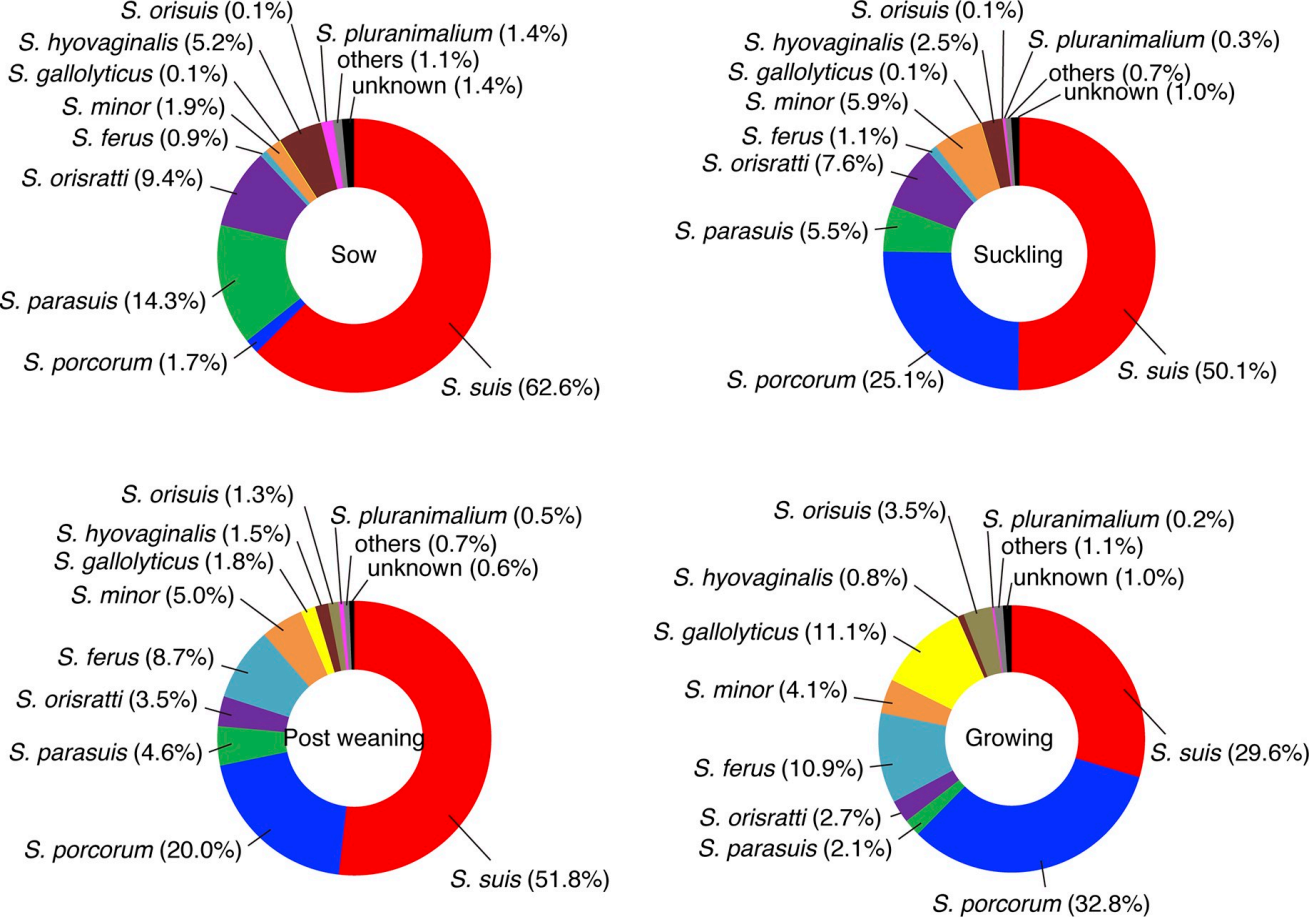
Comparison of species distribution in the genus *Streptococcus* between different growth stages of the pigs. Twenty different species were identified. The top 10 streptococcal species are depicted in different colors. The remaining 10 species are indicated as “others.” The other OTUs identified as *Streptococcus* were not matched to the sequences of the original database; these are indicated as “unknown”.

To analyze the composition of the microbiota in detail, we divided the saliva samples into four groups, based on the age of pigs: suckling piglets, their mothers (i.e., sows), post-weaning piglets, and growing piglets. The definition of these piglets is described in the “Materials and methods” section. *Streptococcus* shared the largest proportion in the saliva of post-weaning and growing piglets because *Streptococcus* shared 16.9%, 18.2%, 19.4%, and 9.9% of the microbiota of saliva from suckling, post-weaning, growing piglets, and sows, respectively (S3 Fig). A customized database comprising all available sequence data that were assigned to the genus *Streptococcus*, including 93 distinct species, was constructed and the OTUs that were recognized as *Streptococcus* were divided at the species level. From this analysis, 20 streptococcal species were identified; the top 10 are depicted in Fig 5. Among these species, *S*. *suis* was detected in saliva of all healthy pigs and was the most abundant in the saliva of suckling piglets (50.1%), post-weaning piglets (51.8%), and sows (62.6%). In the saliva from growing piglets, *S*. *porcorum* (32.8%) was the most abundant and *S*. *suis* (29.6%) was the second most abundant. *S*. *porcorum* was also abundant in the saliva at the other growth stages; however, it shared only 1.7% abundance in the saliva of sows. Based on the proportions of the genus *Streptococcus* in the microbiota, the proportions of *S*. *suis* were estimated as 8.4%, 9.4%, 5.7%, and 6.2% among the total bacteria in the saliva of suckling piglets, post-weaning piglets, growing piglets, and sows, respectively. A new taxon, *Streptococcus parasuis* (which was recently removed from the taxon *S*. *suis*), was also in the top 10 species of *Streptococcus*; however, the proportions ranged 2.1%–14.3%.

Spearman’s rank correlation coefficient revealed that 10 bacterial genera (based on OTUs) in saliva had a significantly positive correlation with *S*. *suis* (Table 2). Four of 10 OTUs also had a significantly positive correlation with *S*. *suis* in swabs of feeder troughs and water dispensers. On the other hand, with a few exceptions, these OTUs showed no correlation in feces and vaginal mucus.

**Table 2:** Bacterial genera (based on OTU) correlated with *S*. *suis* and their correlation values.

Bacterial genus (species) assigned to each OTU	Saliva	Feces	Vaginal mucus	Feeder trough swabs	Water dispenser swabs
*Moraxella* (*pluranimalium*)	0.61**	ND	ND	0.69*	0.82**
*Actinobacillus* (*rossii*)	0.54**	0.11	0.35	0.79**	ND
*Actinomyces* (*denticolens*)	0.48**	ND	ND	ND	ND
*Agromyces* (*insulae*)	0.46**	ND	ND	0.09	ND
*Streptococcus* (*minor*)	0.43**	ND	0.2	0.51	0.74**
*Rothia* (*endophytica*)	0.42**	ND	ND	0.7*	0.28
*Streptococcus* (*equinus*)	0.41**	ND	ND	0.14	ND
*Selenomonas* (*bovis*)	0.41**	ND	ND	ND	ND
*Haemophilus* (*parasuis*)	0.4**	ND	ND	ND	ND
*Moraxella* (*porci*)	0.4**	ND	ND	ND	ND

Bacterial species assigned as the best hit in the BLASTN search of each OTU are shown in the parenthesis for the reference. The label “ND” indicates that no data (i.e., OTUs) exist in the respective samples for correlation analysis.

**p<0.01

*p<0.05.

### Comparison of the microbiota between different sample groups

**Fig 6:**
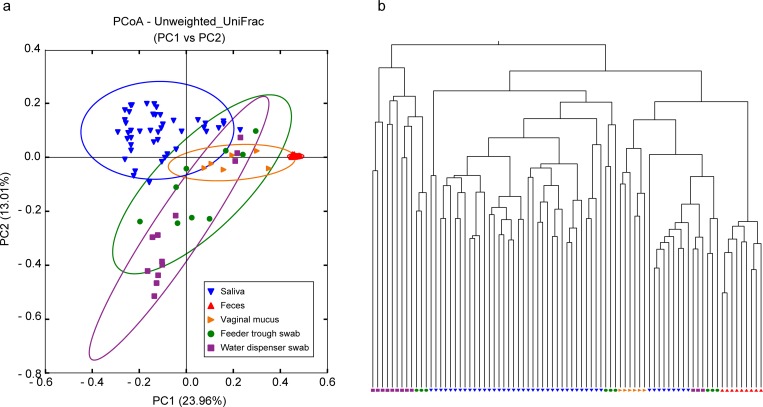
Comparison of microbiota among different sample groups by using β diversity. (a) PCoA measured using unweighted UniFrac distance matrices. The colored ovals show 95% confidence intervals calculated by R. The inset at the lower right shows the symbols and the corresponding samples. (b) The relationship among the microbiota of samples is presented by hierarchical cluster analysis. Symbols at the bottom of the chart are described in the inset in image (a).

Microbial diversity between two or more samples was measured by using β diversity. PCoA and hierarchical cluster analysis can be used to visually demonstrate similarity and the association of the composition of microbiota (i.e., β diversity). A higher β diversity indicates a larger difference in species identity between the respective microbiota, and it displays differences in the composition of both major and minor bacterial taxa. The β diversity among different sample groups was compared by using PCoA and is depicted in Fig 6A. The saliva samples were clustered and were separated from vaginal mucus and feces. The environmental samples were also separated from the saliva samples, but the swabs of feeder troughs were relatively close to the saliva samples. Hierarchical cluster analysis showed that samples were divided into three major groups (Fig 6B). The first group consisted of swabs of feeder troughs and water dispensers. The second group involved most of the saliva samples and a few swabs of feeder troughs. The third group was further divided into three subgroups. Samples of vaginal mucus and feces were separated into two subgroups, and the remaining subgroup consisted of some saliva samples with a few swabs of feeder troughs and water dispensers.

## Discussion

The gut microbiota of pigs has been described in some research papers [35–37]; however, the microbiota of other parts of the pig’s body such as saliva and vaginal mucus and the pig farm environment have not been fully elucidated. In this study, we used 16S rRNA gene amplicon sequencing to show the overall roundup of microbiota in relation to the presence of *S*. *suis* in samples collected from the pig body and from the environment of pig farms in Vietnam where human *S*. *suis* infections have become public health concerns.

Microbial diversity showed the lowest number of common OTUs in saliva and in swabs of water dispensers, which indicated that the microbiota had a high degree of plasticity and that the composition of the microbiota was changeable (Table 1 and Fig 1). On the other hand, the microbiota of feces showed a large number of common OTUs, which suggested that many bacterial genera have the ability to colonize in these niches. This finding was the same at the genus level (Figs 2 and 3A). Regardless of the plasticity in the microbiota of saliva, four genera shared nearly 50% of the microbiota; in particular, *Streptococcus* ranked first and shared 17% of the microbiota (Figs 2 and 3B). This finding was in contrast to the fact that three of four genera other than *Streptococcus* shared very few proportions in the other sample groups. These results suggested that *Streptococcus* has specifically adapted to colonize in the niche, the oral cavity of pigs. Moreover, the predominance of a group of four genera—*Streptococcus*, *Actinobacillus*, *Moraxella*, and *Rothia*—has never been seen in the oral microbiota of other animals, including humans [38–43], which indicates that this is a unique characteristic of the pig oral microbiota.

The LEfSe also highlighted bacterial genera that were significantly abundant in different sample groups. The genus *Streptococcus* had the top LDA score among seven distinct bacterial genera extracted from the saliva samples. Among the seven, three genera ranked in as the top three genera. These results also suggested that *Streptococcus* was specifically adapted for colonization in the oral cavity of pigs, irrespective of the plasticity and changeability of composition of microbiota. In the other sample groups, two genera and three genera in vaginal mucus and in the water dispenser swab, respectively, appeared in top three genera in each sample group. This finding suggested that these genera have also adapted for colonization in these niches of the pig body. In addition, when the samples were divided according to the growth stage of the pigs, the predominance of *Streptococcus* in saliva was also observed. Thus, *Streptococcus* colonizes in pig oral cavity throughout their growth stages.

A recent study demonstrated that 100% of healthy pigs in Japan carried *S*. *suis* in their saliva, and the average proportions of *S*. *suis* to total bacteria in the saliva of different growth stages were estimated as 1%–15% [16]. To confirm whether the same observations would occur in samples from Vietnam, we performed qPCR with *S*. *suis*-specific primer pair and discriminated the genus *Streptococcus* found in the microbiota at the species level. The qPCR data showed a large number of *S*. *suis* cell in saliva samples as compared with other biological or environmental samples, though their abundances were different between samples or growth stages (S3 Table). As shown in Fig 5, in samples collected in Vietnam, *S*. *suis* was the most abundant species among 20 streptococcal species identified in pig saliva in three of four growth stages. These findings indicated that the predominance of *S*. *suis* in pig oral microbiota is a common feature. The estimated average proportions of *S*. *suis* to total bacteria in the microbiota (5.7%–9.4%) were within the range described in the previous study [16]. This result suggests that the high proportion of *S*. *suis* colonization is common in pig saliva, regardless of the difference in climate between countries, method of breeding, and hygiene and facilities of the pig houses.

In the human oral microbiota, the most abundant species are *Streptococcus mitis*, *Streptococcus salivarius*, *Streptococcus sanguinis*, *Streptococcus parasanguinis*, and *Streptococcus oralis*, all of which are commensal [38, 39]. From this point of view, the pig oral microbiota seemed to be unique and exceptional in the fact that one of the major swine bacterial pathogen, *S*. *suis*, was the most abundant. On the other hand, *S*. *suis* also seemed to be an exceptional inhabitant of the oral cavity because this bacterium sometimes may cause severe diseases in its host, although most of the *S*. *suis* isolates from healthy pigs are generally avirulent or showed a low degree of virulence. Moreover, the difference of the most abundant streptococcal species in the oral cavities between pigs and humans clearly demonstrate that the environment for bacterial growth was contrasted with each other. Bacteria always inhabit in niches where they can utilize their favorite nutrients such as oligosaccharides. The oral cavity of pigs may be advantageous for the bacterial growth of particular streptococcal species. An alternative explanation is that *S*. *suis* may have evolved to fit the niche of the pig oral cavity. These speculations are supported by the fact that other bacterial genera (based on OTUs) significantly correlated with *S*. *suis* in saliva, as indicated by Spearman’s rank correlation, were very few in the other sample groups. Thus, the oral cavity of pigs appeared to be distant from other niches and suitable for the growth of these bacteria, as well as *S*. *suis*. In this regard, a few bacterial genera (based on OTUs) appeared in the Spearman’s rank correlation in the swabs of feeder troughs and water dispensers. This finding suggested that these samples were contaminated with the saliva of pigs.

The association of microbiota, based on PCoA and hierarchical analysis, showed that the microbiota of pig saliva was generally distant from that of the other sample groups, which suggests that the microbiota of saliva was different in the major and minor bacterial genera. Moreover, the analyses also depicted that microbiota of pig saliva was somewhat coordinate with those of the swabs of feeder troughs, and in some instances, the water dispenser, which indicated cross-contamination between saliva and the feeder troughs/water dispensers. A previous study [16] also suggested this conclusion; however, the finding in the present study is strong evidence because we have compared the whole genera between different sample groups.

It is generally difficult to determine the source of infection in domestic animals. In many cases, the transmission of infection may occur from the mother to the neonates by vertical transmission during parturition and/or after birth by the fecal-oral route. The symptoms of swine *S*. *suis* infection often appear in post-weaning piglets; therefore, the infection may occur before that stage. The fact that *Streptococcus* was the top genus in the saliva of pigs and *S*. *suis* was the most abundant species in this genus strongly suggested that saliva was the most probable source of *S*. *suis* infection. On the other hand, the aforementioned findings that 100% of pigs contained *S*. *suis* in their saliva, owing to the advantageousness of this niche for the growth of the bacteria, demonstrates that it would be difficult to eliminate this bacterium from healthy pigs. Therefore, to minimize occupational risk, workers handling pigs should take precautions to avoid direct contact with pig saliva. However, the degree of virulence in *S*. *suis* varies among serotypes and strains, and the most virulent serotype, serotype 2, was not detected in any healthy pigs, except for few cases in our previous study [1, 16, 44, 45]. The elimination of a highly virulent type from sow candidates and inhibition of transmission via saliva from sows to piglets may reduce the occurrence of *S*. *suis* infection in post-weaning piglets.

## Supporting information

S1 Fig The phylogenetic tree of 909 representative sequences. (TIF)

S2 Fig The rarefaction curves are presented, based on observed OTUs (a) and Shannon diversity index (b).
(TIF)

S3 Fig Genera distribution in the microbiota. Only the bacterial genera that shared >2% abundance are indicated by different colors, and the genera that shared <2% abundance are collected and then indicated by gray bars. Prefixes of the bacterial names indicate the following ranks: “f” for family, and “g” for genus.
(TIF)

S1 Table Information of 81 samples collected in this study. (XLSX)

S2 Table Information for species assignment in the genus *Streptococcus*. (XLSX)

S3 Table The cell number of total bacteria and *S. suis* estimated by qPCR. (XLSX)
