# Supplementary figures and images for: Characterization of pig saliva as the major natural habitat of Streptococcus suis by analyzing oral, fecal, vaginal, and environmental microbiota
Source: PLoS One. 2019 Apr 24;14(4):e0215983. doi: 10.1371/journal.pone.0215983 (PMC6481863; doi:10.1371/journal.pone.0215983)

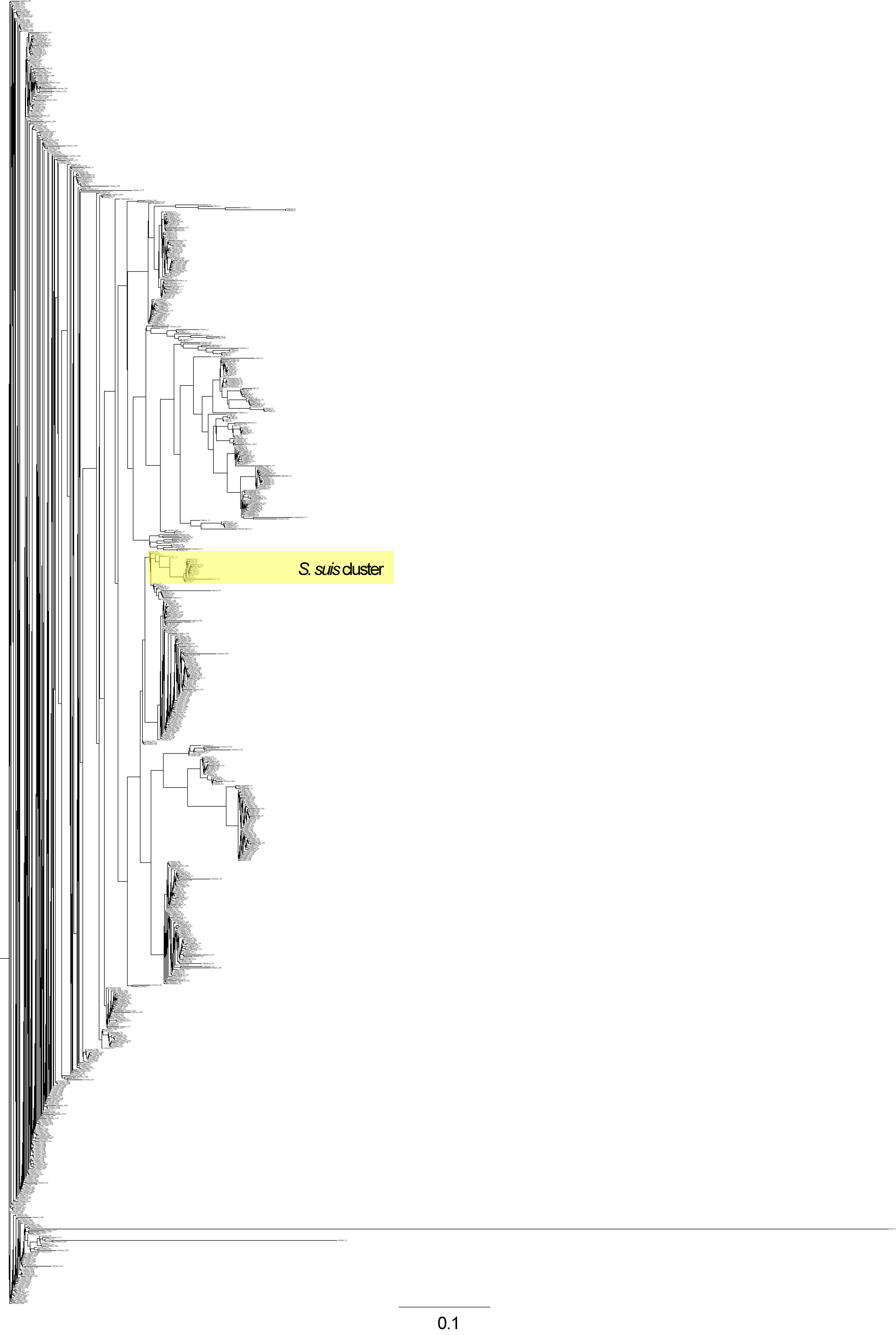

Supplement: S1 Fig — (TIF) [file pone.0215983.s001.tif]

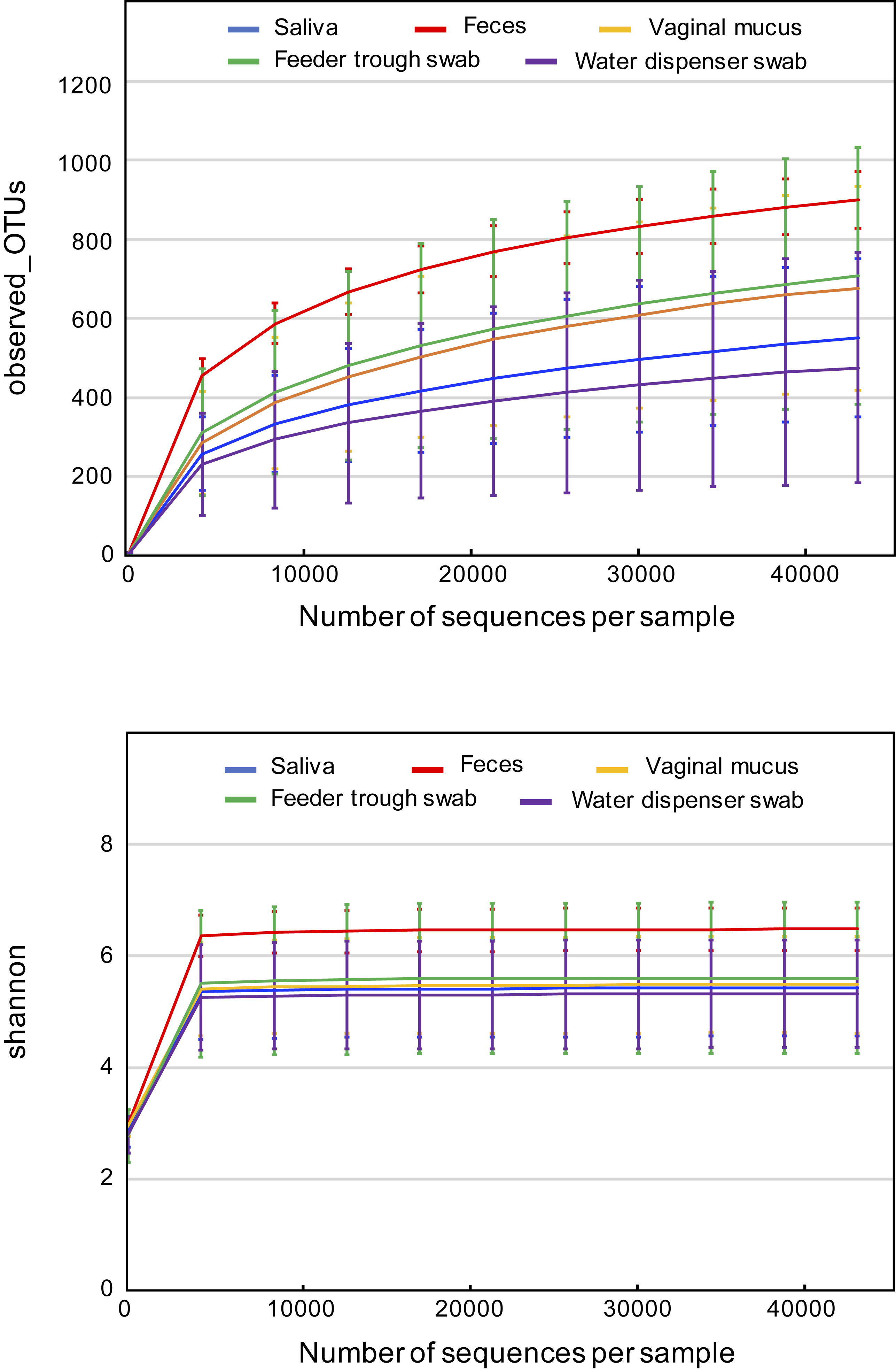

Supplement: S2 Fig — The rarefaction curves are presented, based on observed OTUs (a) and Shannon diversity index (b). (TIF) [file pone.0215983.s002.tif]

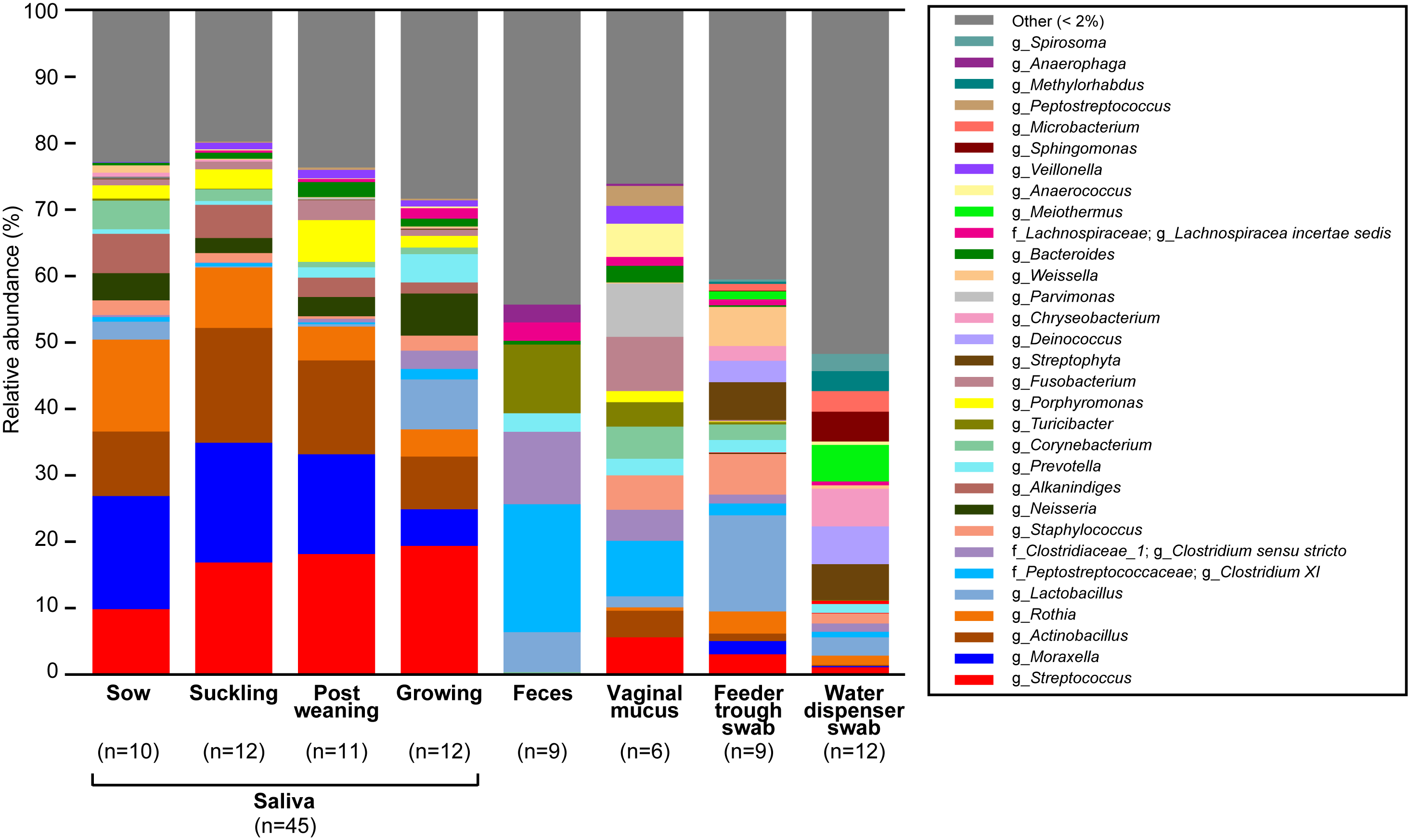

Supplement: S3 Fig — Only the bacterial genera that shared >2% abundance are indicated by different colors, and the genera that shared <2% abundance are collected and then indicated by gray bars. Prefixes of the bacterial names indicate the following ranks: “f” for family, and “g” for genus. (TIF) [file pone.0215983.s003.tif]
